# Connective tissue growth factor-targeting DNA aptamer suppresses pannus formation as diagnostics and therapeutics for rheumatoid arthritis

**DOI:** 10.3389/fimmu.2022.934061

**Published:** 2022-08-05

**Authors:** Gan Wu, Can Liu, Ben Cao, Zelin Cao, Haige Zhai, Bin Liu, Shengwei Jin, Xinyu Yang, Chen Lv, Jianguang Wang

**Affiliations:** ^1^ Department of Anesthesia and Critical Care, The Second Affiliated Hospital and Yuying Children’s Hospital of Wenzhou Medical University, Wenzhou, China; ^2^ Department of Biochemistry, School of Basic Medical Sciences, Wenzhou Medical University, Wenzhou, China; ^3^ Department of Medicinal Chemistry, School of Pharmaceutical Sciences, Wenzhou Medical University, Wenzhou, China; ^4^ Department of Orthopedics, The First Affiliated Hospital of Wenzhou Medical University, Wenzhou, China

**Keywords:** aptamer, connective tissue growth factor (CTGF), rheumatoid arthritis (RA), pannus, thrombospondin 1 (TSP1)

## Abstract

Connective tissue growth factor (CTGF) has been recently acknowledged as an ideal biomarker in the early disease course, participating in the pathogenesis of pannus formation in rheumatoid arthritis (RA). However, existing approaches for the detection of or antagonist targeting CTGF are either lacking or unsatisfactory in the diagnosis and treatment of RA. To address this, we synthesized and screened high-affinity single-stranded DNA aptamers targeting CTGF through a protein-based SELEX procedure. The structurally optimized variant AptW2-1-39-PEG was characterized thoroughly for its high-affinity (KD 7.86 nM), sensitivity (minimum protein binding concentration, 2 ng), specificity (negative binding to other biomarkers of RA), and stability (viability-maintaining duration in human serum, 48 h) properties using various biochemical and biophysical assays. Importantly, we showed the antiproliferative and antiangiogenic activities of the aptamers obtained using functional experiments and further verified the therapeutic effect of the aptamers on joint injury and inflammatory response in collagen-induced arthritis (CIA) mice, thus advancing this study into actual therapeutic application. Furthermore, we revealed that the binding within AptW2-1-39-PEG/CTGF was mediated by the thrombospondin 1 (TSP1) domain of CTGF using robust bioinformatics tools together with immunofluorescence. In conclusion, our results revealed a novel aptamer that holds promise as an additive or alternative approach for CTGF-targeting diagnostics and therapeutics for RA.

## Introduction

Rheumatoid arthritis (RA) is a chronic systemic autoimmune disorder, typically characterized by persistent synovitis and aggressive pannus formation, which leads to bone and cartilage injury (1–4). To date, although the combination of biologic and conventional synthetic disease-modifying anti-rheumatic drugs (DMARDs) has improved the quality of life of patients with RA, disease activity score-defined early remission is only achieved in less than 50% of treated patients, and a mere 4% remain in drug-free remission during 4-year follow-up (5). Therefore, new and specific therapeutic targets are attracting attention but remain to be further explored in RA.

Recent studies have found that connective tissue growth factor (CTGF), also known as CCN family protein 2, is an ideal biomarker, especially in the early course of RA (6, 7). Apart from its known functions regarding cell adhesion and fibrosis, the angiogenic role of CTGF, which contributes to pannus formation in RA (8, 9), is attracting attention. In our previous proteomic research, CTGF was significantly elevated in the fibroblast-like synoviocytes (FLS) of 50 patients with RA compared with that in 50 healthy controls (10). Furthermore, we confirmed a similar increase in CTGF levels in the serum samples of 98 patients with RA and identified the diagnostic value of serum CTGF with sensitivity, specificity, and AUC of ROC curve at 0.86, 0.92, and 0.92, respectively, at the optimal cutoff value of 88.66 pg/ml (11). In addition, it was reported that CTGF promotes articular damage by increasing the proliferation of FLS in RA (12). Importantly, anti-CTGF mAb treatment prevented the progression of arthritis in CIA mice, suggesting the potential of CTGF as a new target for RA treatment (13). In summary, CTGF serves as a specific biomarker for early diagnosis and is also an important target for the treatment of RA.

However, current approaches for detecting CTGF are limited by traditional immunological methods, such as ELISA (14), immunohistochemistry (15), and immunosensor (16), which are costly or limited in use. By contrast, to antagonize CTGF for therapeutic purposes, an antibody is mainly used for antifibrosis while missed in RA treatment, to say nothing of its major disadvantages in terms of immunogenicity, unsatisfactory storage, and long-term exploitation (17–19).

In this case, aptamers are seemingly preferable alternatives, which have emerged as appealing biologicals for clinically practical applications (20, 21). Aptamers are single-stranded DNA or RNA oligonucleotides that can be designed to specifically and clinically inactivate relevant molecules, which are generated through a process termed “systematic evolution of ligands by exponential enrichment” (SELEX) (22–24). In recent years, aptamer screening has been developed for a wide range of agents, including organic molecules, proteins, viruses, bacteria, and whole cells, across fields, such as drug development, medical imaging, and biological detection (25–30). Comparatively, longer shelf life, immunogenicity-free inherence, higher stability, and lower synthesizing cost give aptamers several advantages over antibodies (31, 32). However, the development of clinically effective therapeutic aptamers has lagged far behind that of therapeutic antibodies (20, 33). To date, Li et al. (34) and Gao et al. (35) have successively developed aptamers targeting CTGF; however, the former was limited in the screening procedure while the latter paused at its diagnosis application. Thus, neither advanced to the therapeutic application of CTGF-targeting aptamer, particularly in RA.

In this study, we sought to generate novel CTGF-targeting DNA aptamers that suppress pannus formation for use as both diagnostics and therapeutics for RA. In particular, functional experiments and animal models were employed for the therapeutic effect test, indicating practical significance for therapeutic prospects.

## Materials and methods

### Single-stranded DNA library construction

We constructed a library of single-strand DNAs with a whole length of 76 nt, consisting of a 36-nt randomized central sequence flanked on both ends by 20-nt fixed primer sequences (5΄-TTCAGCACTCCACGCATAGC [N]36 CCTATGCGTGCTACCGTGAA-3΄, N = A, T, G, C). The synthesis of the aptamers and the initial library was outsourced to Sangon Biotech Co., Ltd. (Shanghai, China). Quick renaturation following denaturation prepared the library for use. The sequence of AptW2-1-39 was as follows: 5΄-AGGCCGGGAGGGTACCCATTGATGGTGTGATGACTGCCT-3΄.

### SELEX procedure

**Figure 1:**
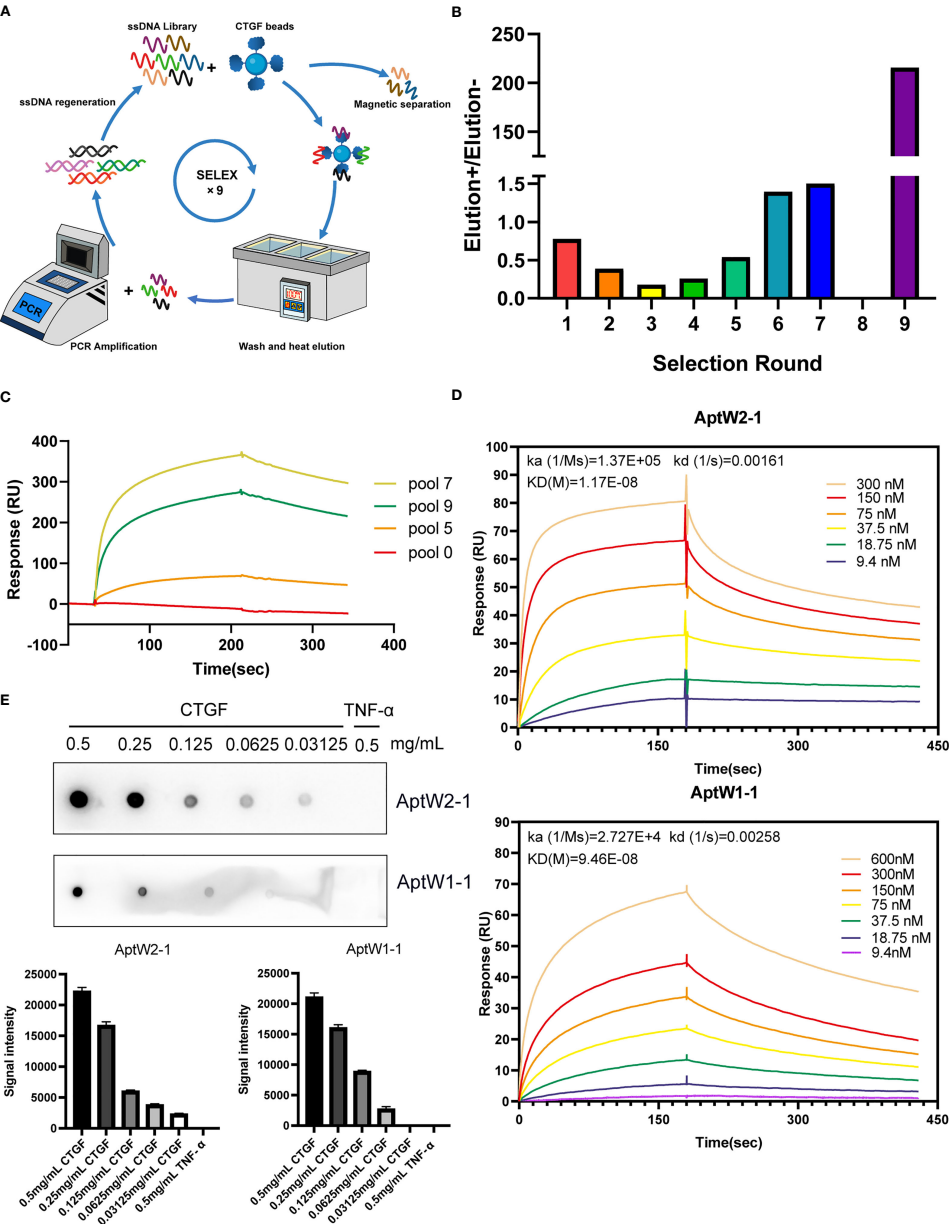
SELEX strategy for CTGF aptamer screening. **(A)** Schematic diagram of the SELEX procedure. **(B)** Output ratio of the forward selection/counter selection in successive rounds assayed using qPCR. **(C)** SPR assay of enriched aptamer pools targeting CTGF. **(D)** SPR assay of AptW2-1 and AptW1-1 targeting CTGF. **(E)** Dot blot assay of AptW2-1 and AptW1-1 targeting CTGF and TGF-β, respectively.

Iterative rounds of the selection and amplification of ssDNA aptamers were performed to obtain specific aptamers for CTGF. Briefly, bovine serum albumin (BSA) and CTGF proteins (R&D Systems, Minneapolis, MN) were coupled with carboxyl magnetic beads, denoted as MB-BSA and MB-CTGF, respectively. In the first round of SELEX, 100 µl of 5 µM ssDNA library was first incubated with MB-BSA for counter selection and then with MB-CTGF for positive selection. The ssDNA products were then separated from the CTGF by heating and eluting. After PCR amplification and ssDNA regeneration, the ssDNA products were enriched for a successional loop of screening, which circulated for nine rounds in total. Additionally, serum samples from healthy people were added in counter selection starting from the eighth round. Quantification of the ssDNAs was assayed using real-time PCR in each round of SELEX. “Elution+” and “Elution-” (**Figure 1B**) indicate quantification of ssDNAs after positive elution (bound by CTGF protein) and counter elution (bound by BSA, serum), respectively, assayed using qPCR in each round of SELEX. In addition, the screened ssDNA products were subjected to high-throughput sequencing (HTS) at Sangon Biotech Co. Ltd. (Shanghai, China). Clustal X software was used for sequence comparison and classification.

### Surface plasmon resonance assay

The binding of the aptamers obtained to CTGF was investigated using a Biacore T200 instrument (GE Health Care Life Sciences, Uppsala, Sweden). Briefly, CTGF was coupled with the second channel of the CM5 chip (GE Healthcare) while the first channel remained vacant, only activated and blocked, thereby denoted as a negative control. For binding analysis, the aptamers were diluted with running phosphate buffered saline (PBS) to a series of concentrations (600 nM, 300 nM, 150 nM, 75 nM, 37.5 nM, 18.75 nM, and 9.375 nM) and pumped over the chip surface for 3 min at a flow rate of 10 µl/min, in the order of descending concentration. The pure running buffer was added for another 5 min to continue the dissociation followed by NaCl injection (1 M) for 30 s at 30 µl/min to achieve regeneration.

The binding of the aptamers to RA-related cytokines and different deletion mutants of CTGF was explored using the same instrument with an SA chip coupled with a biotinylated aptamer. In this case, the samples were pumped over the surface of the chip for 120 s at a flow rate of 10 µl/min. The data collected were evaluated using a Biacore T200 Evaluation Software version 3.2.1.

“Response” represents the response signal value of the SPR instrument, which is a refractive index sensor whose response value reflects the change in SPR angle. A 1 RU change in “response” is approximately equivalent to a 1 pg/mm^2^ change in the concentration of the analyte binding to the surface of the chip.

### Dot blot assay

For binding analysis, the protein was diluted into a series of concentrations and then spotted (1 µl/dot) onto a nitrocellulose membrane using a narrow-mouth pipette tip. The membrane dried naturally and was blocked using 10% BSA (2 h at room temperature). Subsequently, the membrane was incubated with biotinylated aptamers (1 μM) for another 2 h followed by HRP-labeled streptavidin for 30 min at room temperature. The ImageQuantTMLAS 4000 digital imaging system (GE Health Care Life Sciences) was applied for photography.

### Aptamer stability assay

To assess the stability of the acquired products, AptW2-1 (0.5 µl of 10 µM) was incubated with 10 µl of plasma at 37°C. By contrast, an equivalent aptamer was also added to 10 μl of PBS as a control. After 0 h, 6 h, 12 h, 24 h, and 48 h of incubation, the samples were collected and incubated at 65°C for 10 min to inhibit nuclease activity. Finally, the samples were run on 3% agarose gel (Sigma-Aldrich, Saint Louis, USA) in 1 × TAE buffer and visualized with GelRed (Sigma-Aldrich). The lanes were quantified and analyzed using Image Lab software.

### Cell proliferation assay

Cell proliferation activities were examined using human umbilical vein endothelial cells (HUVECs). The cells were seeded onto 96-well plates (1 × 10^4^ cells/well) for 24 h and treated with a fresh culture medium with the addition of PBS, CTGF (100 ng/ml), CTGF (100 ng/ml)+AptW2-1-39-PEG (100 nM), CTGF (100 ng/ml)+AptW2-1-39-PEG (200 nM), or CTGF (100 ng/ml)+AptW2-1-39-PEG (500 nM) for 0 h, 12 h, 24 h, 36 h, or 48 h at 37°C. The proliferative capacity of HUVECs was determined using a CCK8-based cell proliferation and viability assay system according to the manufacturer’s instructions (Dojindo, Kumamoto, Japan).

### Transwell assay

A transwell assay was performed to evaluate the migration of HUVECs. Briefly, 600 µl of 20% fetal bovine serum medium with or without recombinant human CTGF (100 ng/ml) was added to each lower chamber of the 24-well transwell inserts (8.0 μm pore size; Corning, Corning, NY). The transwell inserts (upper chambers) were then placed in the wells (lower chambers). HUVECs (5 × 10^4^) in 300 µl of serum-free medium (containing PBS, 100 nM, 200 nM, or 500 nM AptW2-1-39-PEG) were added to each upper chamber and incubated for 12 h. Nonmigrating cells were removed with a cotton swab. Cells that migrated to the lower phase of the upper chamber were then fixed in methanol for 30 min and stained with crystal violet (1 mg/ml; Fluka) for 30 min at room temperature. The excess stain was removed with water, and the chambers were air-dried. Images were obtained under the microscope, and the cell number was quantitated using ImagePro software.

### Endothelial tube formation assay

An endothelial tube formation assay was performed to evaluate angiogenic activity *in vitro*. Briefly, Matrigel (BD, New Jersey, USA) was diluted with Dulbecco’s modified Eagle’s medium (DMEM) at a ratio of 1:1 and then used to coat 24-well plates (200 µl/well) under incubation at 37°C for approximately 1 h to promote gelling. HUVECs were first starved for 24 h and then resuspended in DMEM after centrifugation. Next, the HUVECs were added to each well with PBS, CTGF (100 ng/ml), CTGF (100 ng/ml)+AptW2-1-39-PEG (100 nM), CTGF (100 ng/ml)+AptW2-1-39-PEG (200 nM), or CTGF (100 ng/ml)+AptW2-1-39-PEG (500 nM) and then incubated in a humidified incubator at 37°C with 5% CO_2_. After 6 h of incubation, the plates were observed and captured under a microscope. Tube-like structures in each well were evaluated using the number of intersections among branches of the endothelial cell networks in the whole field.

### Chick chorioallantoic membrane assay

To assess angiogenic activity *in vivo*, embryonated chicken eggs were incubated at 37°C and 40–60% humidity; silicone rings were placed on the CAM surface. All eggs were randomly divided into five groups and treated with PBS, CTGF (100 ng/ml), CTGF (100 ng/ml)+AptW2-1-39-PEG (100 nM), CTGF (100 ng/ml)+AptW2-1-39-PEG (200 nM), or CTGF (100 ng/ml)+AptW2-1-39-PEG (500 nM) on day 7. Neonatal blood vessels in the embryos were observed 3 days later under a stereomicroscope. For analysis, Image J 2.43 was used to assess the vascular and CAM areas. The percentage of the angiogenic area was calculated using the following equation:

% Angiogenic areas = vascular areas/CAM areas × 100%.

### Aptamer binding assay to murine CTGF

Serum collected from a healthy mouse was subjected to TCA acetone precipitation. The precipitated serum protein was redissolved and incubated overnight with a biotinylated aptamer at 4°C. The following day, magnetic beads coupled with streptavidin (Thermo Fisher Scientific, Waltham, Massachusetts) were added and rotated for incubation at room temperature for 2 h. Through magnetic isolation, aptamer-binding proteins were collected and then detected with anti-CTGF antibody using Western blotting.

### Collagen-induced arthritis mice model establishment and histopathology evaluation

DBA/1 mice (males, 8 weeks old) weighing 18–20 g each were purchased from SLAC Laboratory Animal Co. (Shanghai, China). All mice were raised in a specific-pathogen–free room at the Laboratory Animal Center of WMU and housed in cages (five per cage) kept at 22–26°C and 60–65% humidity on a regular 12-h light/dark cycle (light, 8:30–20:30). All procedures in the animal experiments were endorsed by the Institutional Animal Care and Use Committee of Wenzhou Medical University.

Arthritis was induced through the CIA model establishment in strict accordance with the procedure. Briefly, for the first immunization, type II collagen (Chondrex, Redmond, WA, USA; 2 mg/ml, 100 μg/each) and complete Freund’s adjuvant (Sigma-Aldrich) were equally mixed and fully emulsified in an ice bath and then injected into the back of the mice on day 0. For the second immunization, type II collagen and incomplete Freund’s adjuvant were prepared similarly and injected into the skin at multiple points at the tail root on day 21, avoiding the first immunization site. Meanwhile, we injected AptW2-1-39-PEG into the knee joints (1 μg/joint) and enterocoelia (100 μg/mouse) of the mice once every other day starting from day 21 to day 48. On day 49, the mice were sacrificed, and their joint tissues and serum samples were harvested for analysis (**Figure 5C**).

The limbs of the sacrificed mice were fixed in 4% paraformaldehyde and decalcified in 50 nM ethylenediaminetetraacetic acid solution for hematoxylin and eosin (H&E) staining to show the morphology of the joints. Immunohistochemical (IHC) staining was used to detect the proliferation of joint synovial fibroblasts in the CIA mice. Briefly, knee joint tissue sections were blocked with BSA and incubated overnight at 4°C with a primary antibody against Ki67 (AF0198, affinity, USA) followed by incubation with HRP-labeled secondary antibody for 1 h at room temperature. The 3,3′-diaminobenzidine substrate was used for visualization. High-resolution images were captured using an Eclipse 80i microscope (Nikon, Tokyo, Japan).

### Enzyme-linked immunosorbent assay

The concentrations of IL-1β, tumor necrosis factor alpha (TNF-α), IL-6, and IL-10 in the serum samples of the CIA mice were detected using ELISA kits (R&D Systems) (presented in **Figure 5G**). As per the manufacturer’s instructions, the specimens were diluted to 50 μl (1:20) and measured at an optical density (OD) of 450 nm using spectrophotometry.

To evaluate the immune response, we isolated peripheral blood mononuclear cells (PBMCs) of healthy people and incubated them in 24-well plates at 1 × 10^6^ cells per well. We added AptW2-1-39-PEG with a gradient of concentrations (0 µM, 0.5 µM, 1 µM, and 2 µM) to an RPMI 1640 medium and incubated the human PBMCs for 24 h. Subsequently, we detected the concentrations of TNF-α and IFN-α in the culture supernatant using ELISA kits (R&D Systems) (presented in **Figures S5A**, **B**). We additionally injected AptW2-1-39-PEG into the knee joints (1 μg/joint) and enterocoelia (100 μg/mouse) of the healthy DBA/1 mice once every other day. Serum samples of the treated mice were collected on day 14 and day 28 after injection for detection of total IgM and IgG using ELISA (presented in **Figure S5C**).

### Immunofluorescence

To identify the specific aptamer-binding domain of the CTGF, glass slides of HEK293T cells that were anchored to different deletion mutants of CTGF were incubated with a Cy5-labeled AptW2-1-39-PEG followed by a primary anti-FLAG antibody (1:200 dilution) and then a corresponding secondary antibody (1:400 dilution). Finally, the Eclipse 80i microscope (Nikon, Tokyo, Japan) was used for imaging.

### Homology modeling and molecule docking

Homology modeling of the TSP1-CTCK of CTGF was performed using Modeller 9.25 (36). The sequence of the TSP1 domain was downloaded from the UniProt database (accession number: P29279). In this study, we modeled the TSP1-CTCK structure based on the multitemplate technique. The crystal structures of TSP1-CCN3 (PDB ID: 6RK1) (37) and human Gremlin-1 (PDB ID: 5AEJ) (38) were used as a model template. For the specific modeling steps, see the official Modeller tutorial. After modeling, 10 conformations were obtained, and the conformations with the lowest DOPE score were selected for further study.

To obtain a reasonable TSP1-CTCK/AptW2-1-39 complex, the protein-DNA docking method was adopted to search for the binding conformation. The docking work was performed using the HDOCK server (39). In the end, the top-ranking model was selected for molecular dynamics (MD) simulation for structure optimization and stability evaluation.

### Molecular dynamics simulation

Before the simulation, a protein/DNA system was prepared using the LEaP module of the Amber16 package (40). The Amber force field ff14SB (41) and DNA force field OL15 (42) were applied for describing protein and DNA, respectively. Then, the complex system was immersed into a rectangular periodic box of pre-equilibrated TIP3P water with at least 10 Å distance around the complexes. Finally, appropriate numbers of sodium counter ions were added to maintain the electroneutrality of the simulation system. MD simulations were performed using the pmemd module in AMBER16 package. During the MD simulations, periodic boundary conditions were employed, and the direct space interaction was calculated using the particle mesh Ewald method with a long-range electrostatic interaction (43). All bonds involving hydrogen atoms were constrained with the SHAKE algorithm allowing an integration time step of 2 fs. The MD simulations revealed that the complex model of cluster 1 was the most stable, which is visually analyzed in the *Results* section. PyMOL was used for the visualization and analysis of the aptamer–protein interface.

### Statistical analysis

All statistical analyses were calculated and graphed using GraphPad Prism 8 (GraphPad Software, Inc, La Jolla, CA). The Shapiro–Wilk method was used to test whether the data were normally distributed. The Levene method was used to test the homogeneity of the variance. The one-way analysis of variance test with *post hoc* contrasts by Tukey’s test was applied to compare the means of the multiple groups. The Kruskal–Wallis and Mann–Whitney nonparametric tests were used to compare interassay differences in the data that did not meet the normal distribution or the homogeneity of the variance. *p*-values < 0.05 were considered significant. All data are presented as mean ± SD.

## Results

### Selection of high-affinity aptamers targeting CTGF

We established an ssDNA library totaling 1 × 10^15^ randomized single nucleotide chains. Next, a protein-based SELEX procedure was employed for high-affinity aptamer screening targeting CTGF, as demonstrated in the flowchart (**Figure 1A**). To raise targeting specificity, counter elution using magnetic beads coupled with BSA was applied to eliminate nonspecific ssDNA anterior to forward elution targeting CTGF in each round. Additionally, serum samples from healthy people were added in a counter elution from the eighth to ninth round to further improve target specificity. The quantification results showed that the output of the forward selection began to surpass that of the counter selection from the fourth round, suggesting the enrichment of ssDNAs targeting CTGF. In the subsequent rounds, the quantity of CTGF-binding ssDNAs continued to rise, which topped out observably at the end of the ninth round (**Figure 1B**). Owing to the addition of healthy serum, which excluded a mass of nonspecific ssDNAs, a slight decline in affinity with CTGF was observed in pool 9 but with an increase in specificity compared with that of pool 7. Roughly, affinity detection of selective pools with CTGF indicated a steady climb during the screening process (**Figure 1C**). Furthermore, by comparing the top 100 sequences from the final products based on HTS analysis (detailed in **Figure S1**), we identified multiple groups of similar sequences, which were finally extracted into six groups, with high intra-group consistency (detailed in **Figure S2**). From these grouped sequences, we selected a total of 18 strands for SPR detection (detailed in **Figure S3A**). As illustrated, AptW2-1 (KD 11.7 nM) and AptW1-1 (KD 94.6 nM) showed strong binding ability to CTGF (**Figure 1D**) while AptW1-2 (KD 142.1 nM) showed a lower binding efficiency (**Figure S3B**). Additionally, the specific binding between aptamers and CTGF was also verified compared with that of TNF-α using a dot blot assay (**Figure 1E**).

Here, we successfully obtained efficient aptamers with high affinity—AptW2-1 and AptW1-1—targeting CTGF.

### Structural prediction and properties of assays of the screened aptamers targeting CTGF

**Figure 2:**
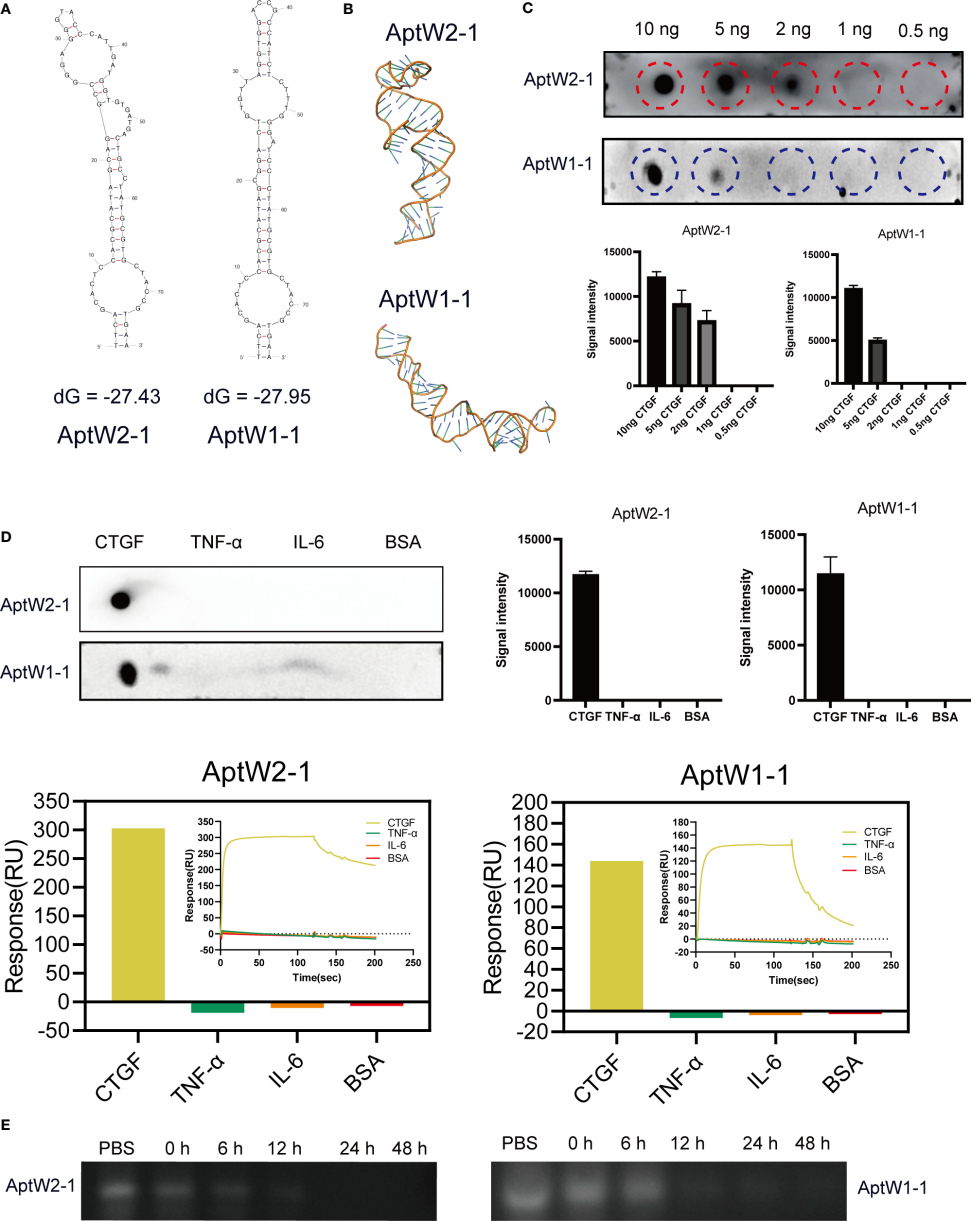
Bioinformatic structural analysis and properties of assays of the aptamers obtained. **(A)** Secondary structural prediction of AptW2-1 and AptW1-1. **(B)** Tertiary structures of the analog images of AptW2-1 and AptW1-1. **(C)** Sensitivity assays of AptW2-1 and AptW1-1 targeting CTGF at gradient concentrations. **(D)** Specificity assays (upper) and SPR assays (lower) of AptW2-1 and AptW1-1 were tested with other RA-related cytokines, including TNF-α and IL-6. **(E)** Stability assays of AptW2-1 and AptW1-1 were incubated with plasma from healthy people.

To predict the secondary and ternary structures of the screened aptamers, we applied “mfold” software (44) and RNAcomposer software (45) for structural analysis. The results showed that AptW2-1 and AptW1-1 possessed fairly stable structures. The most probable secondary structures of AptW2-1 (ΔG = –27.43) and AptW1-1 (ΔG = –27.95) were demonstrated at their lowest free energies (**Figure 2A**). The ternary structures of the pair with minimum free energy were further predicted based on their secondary structures (**Figure 2B**).

For sensitivity assessment, a dot blot assay was used to reflect the minimum protein binding concentration of AptW2-1 and AptW1-1. Briefly, CTGF was diluted into decreasing concentrations of 10 ng, 5 ng, 2 ng, 1 ng, and 0.5 ng and then incubated with the aptamers. As illustrated, AptW2-1 combined with CTGF at the least concentration of 2 ng, and AptW1-1 combined with CTGF at the least concentration of 5 ng, indicating their high binding efficiencies (**Figure 2C**).

For specificity assessment, AptW2-1 and AptW1-1 were also tested with other RA-related cytokines, including TNF-α and IL-6, for binding detection. As illustrated, AptW2-1 and AptW1-1 showed no binding signal with these markers (**Figure 2D**), suggesting the promising potential of the pair for CTGF-specific detection in RA.

For stability assessment, we further simulated according to the conditions of AptW2-1 and AptW1-1 when acting in human plasma. However, the results showed that AptW2-1 and AptW1-1 degraded in less than 12 h when acting in plasma from healthy humans (**Figure 2E**).

Comparatively, AptW2-1 proved to be a more preferable candidate since its affinity and sensitivity were both superior to those of AptW1-1, albeit with inadequate stability, which required further optimization.

### Truncation and PEGylation optimization of AptW2-1 into AptW2-1-39-PEG

**Figure 3:**
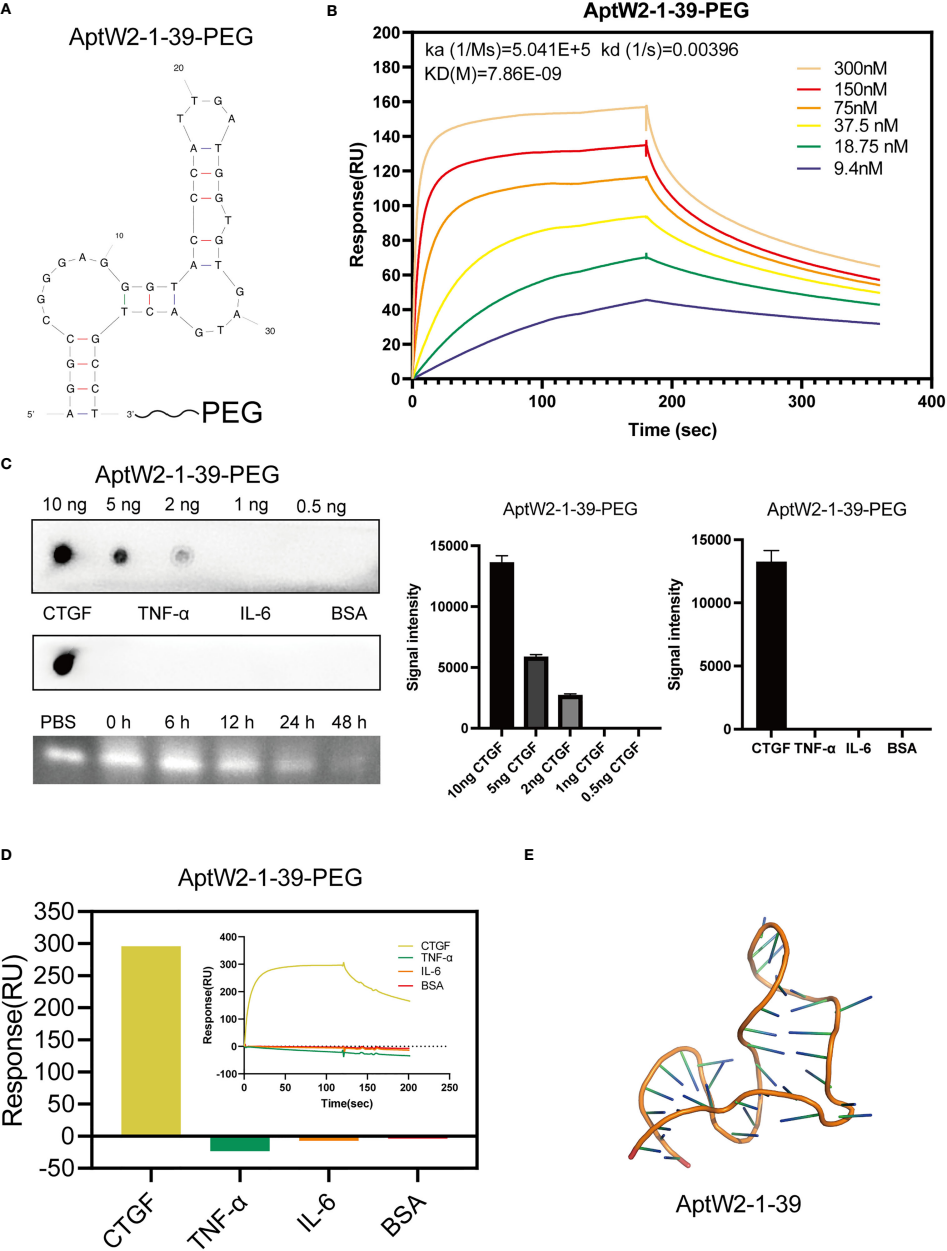
Truncation and PEGylation optimization of AptW2-1 into AptW2-1-39-PEG. **(A)** Truncated conformation of AptW2-1-39-PEG. **(B)** SPR assay of AptW2-1-39-PEG targeting CTGF. **(C)** Sensitivity, specificity, and stability assays of AptW2-1-39-PEG. **(D)** SPR assay of AptW2-1-39-PEG with RA-related cytokines TNF-α and IL-6. **(E)** Tertiary structures of the analog image of AptW2-1-39.

As nucleic acid biopolymers, the *in vivo* therapeutic potency of aptamers is critically limited by their inherent physicochemical characteristics. Therefore, some chemical modifications and conjugations have been developed to improve the pharmacokinetic properties of aptamer-based therapeutics (46). Here, we truncated the nucleotides at both ends of AptW2-1 and kept 39 nt of the central sequence as a reservation for effective combination and added a high-molecular-mass PEG to the 3΄-terminus of AptW2-1 to overcome renal filtration and extend circulation time in a physiological environment (**Figure 3A**). As expected, the optimized AptW2-1-39-PEG showed a substantial increase in both affinity (KD 7.86 nM) (**Figure 3B**) and stability, with high sensitivity and specificity preserved (**Figures 3C, D**). As illustrated, 500 nM of AptW2-1-39-PEG possessed a duration of 48 h, maintaining viability in the plasma of healthy people (**Figure 3C**). Similarly, we performed the tertiary structure prediction of AptW2-1-39-PEG (**Figure 3E**). AptW1-1 was also modified with PEG; however, it was only accompanied by an increase in stability (**Figures S4A–C**).

### AptW2-1-39-PEG suppressed the proliferative and angiogenic activities induced by CTGF

**Figure 4:**
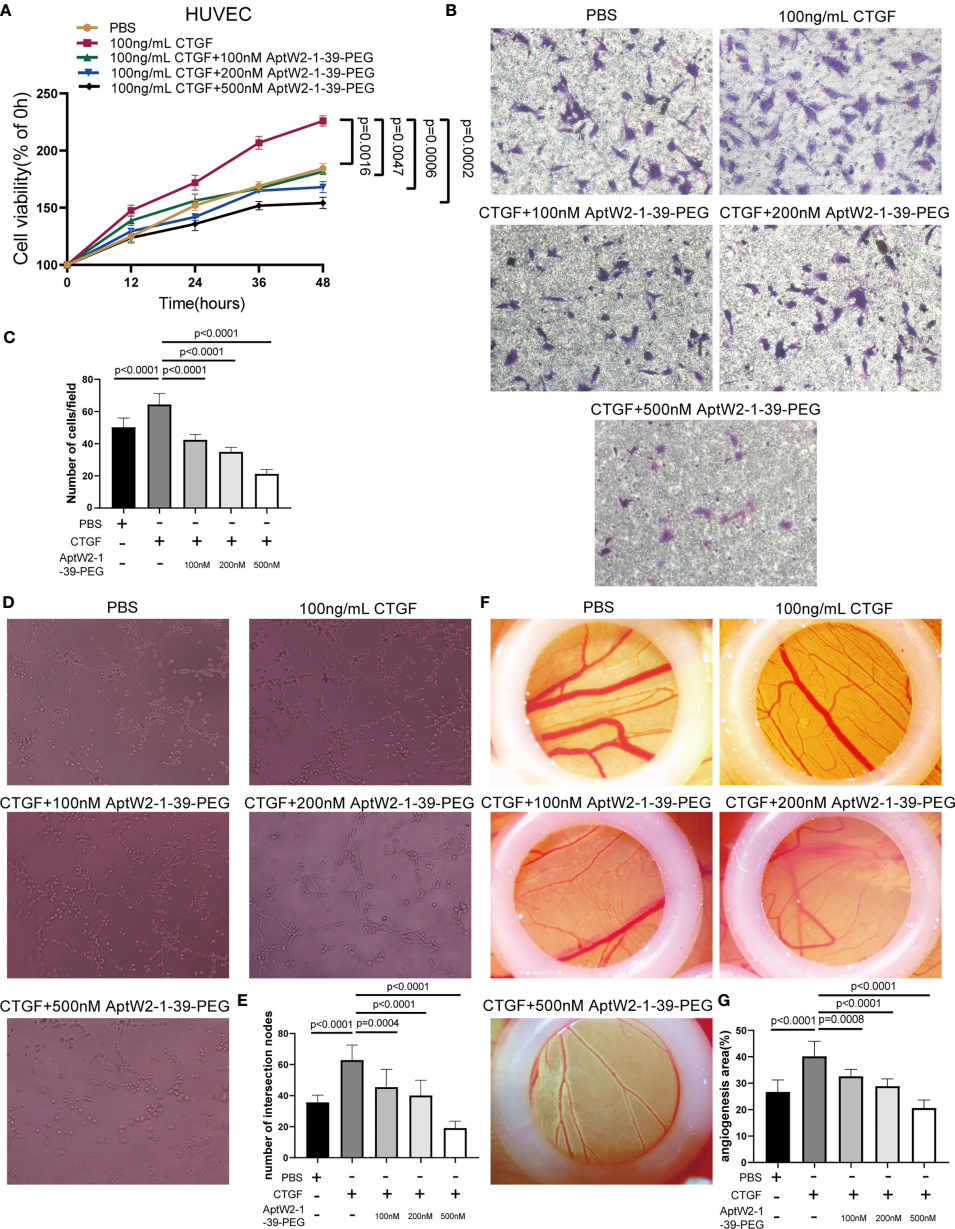
Restrained effect of AptW2-1-39-PEG on the proliferative and angiogenic activities induced by CTGF. **(A)** CCK8 assay. HUVECs were treated with CTGF (100 ng/ml) and AptW2-1-39-PEG (100 nM, 200 nM, and 500 nM) for 12 h, 24 h, 36 h, and 48 h, respectively. The absorbance value of each well at a wavelength of 450 nm was measured. **(B, C)** HUVECs were plated onto the top of the transwell microplates, allowed to migrate for 12 h, and then rinsed, fixed, stained, and counted. **(B)** Images of cells on the undersurface of a filter are shown (original magnification, 200×). **(C)** The number of cells per field in the control and treated cells is shown. **(D, E)** Three-dimensional tube formation assay. HUVECs were treated with CTGF (100 ng/ml) and AptW2-1-39-PEG (100 nM, 200 nM, and 500 nM) for 6 h. **(D)** Tube formation was observed and photographed. **(E)** The number of intersection nodes was calculated in the whole field. **(F, G)** CAM assay. CAM were treated with CTGF (100 ng/ml) and AptW2-1-39-PEG (100, 200, and 500 nM) on day 7. **(F)** Neonatal blood vessels were photographed 3 days later. **(G)** ImageJ was used to assess the vascular and CAM areas. The percentage of the angiogenic area = vascular area/CAM area × 100%. The data are presented as mean ± SD.

Given that CTGF exerted promotion in angiogenesis, which was critical for pannus formation in RA, we further evaluated the effect of AptW2-1-39-PEG on the angiogenic activity induced by CTGF. In our previous study, recombinant human CTGF enhanced the proliferation and migration of HUVECs (10). Here, we verified that proliferation of HUVECs exposed to AptW2-1-39-PEG was significantly decreased according to CCK8 assay, which was dose-dependent and achieved strongest at the concentration of 500 nM (**Figure 4A**). Similarly, AptW2-1-39-PEG decreased the migration of HUVECs in a dose-dependent manner, as shown using the transwell assay (**Figures 4B, C**). The endothelial tube formation and CAM assays were introduced for angiogenic activity evaluation *in vitro* and *in vivo*, respectively. As expected, AptW2-1-39-PEG decreased the number of neonatal tubes compared with the control, as shown using the endothelial tube formation assay (**Figures 4D, E**), while AptW1-1-PEG failed to show such effect (**Figures S4D, E**). Similarly, AptW2-1-39-PEG reduced the generation of microvessels verified using the CAM assay (**Figures 4F, G**). In general, this suggested that AptW2-1-39-PEG blocked the proliferation and angiogenesis induced by CTGF, which provides insight into the pathogenesis of RA.

### AptW2-1-39-PEG relieved joint injury and inflammatory response in the CIA model

**Figure 5:**
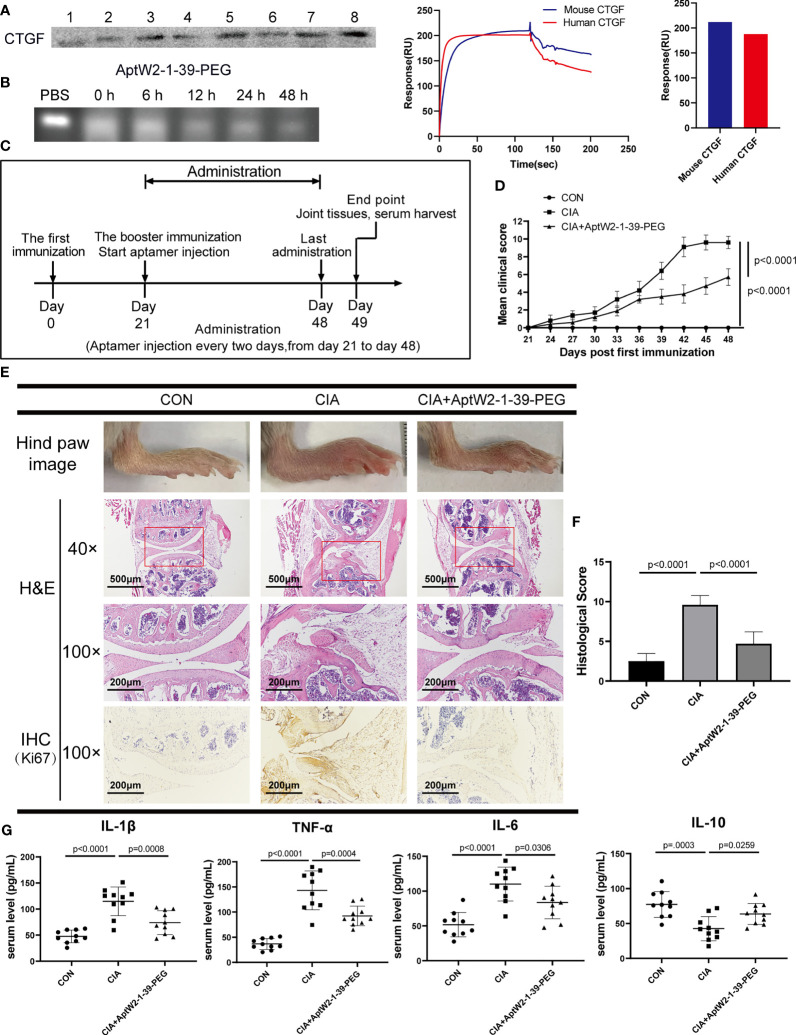
Therapeutic effect of AptW2-1-39-PEG on joint injury and inflammatory response in CIA model. **(A)** Detection of murine CTGF extracted using biotinylated AptW2-1-39-PEG and Western blotting (left). SPR assay of AptW2-1-39-PEG targeting murine CTGF (right). **(B)** Stability assay of AptW2-1-39-PEG incubated with murine plasma. **(C)** Timeline of CIA model establishment treated with AptW2-1-39-PEG (*n* = 10 per group). **(D)** Arthritis clinical scores of the CIA mice. The scoring system was defined as 0 = no evidence of erythema and swelling, 1 = erythema, and mild swelling confined to the tarsals or ankle joint, 2 = erythema and mild swelling extending from the ankle to the tarsals, 3 = erythema and moderate swelling extending from the ankle to the metatarsal joints, and 4 = erythema and severe swelling encompass the ankle, foot, and digits, or ankylosis of the limb. Significance was tested using analysis of variance (ANOVA) of repeated measurement. **(E)** Macroscopic images and histopathology evaluation of the CIA mice. Macroscopic images of the ankles of mice were taken on day 49 before being sacrificed (upper panel). H&E staining of knee joints (40×, 100×, and middle panel). IHC staining of joint synovial fibroblasts (lower panel). **(F)** Semiquantitative scores for inflammatory cell infiltration, synovial hyperplasia, and bone destruction were assessed using H&E staining graded on a scale of 0 (normal) to 3 (severe) for 4 paws in 12. **(G)** The concentrations of the cytokines in the serum of the CIA mice were detected using ELISA. The data are presented as mean ± SD.

The CIA murine model was introduced to evaluate the function of AptW2-1-39-PEG in RA progression. We preliminarily applied biotinylated AptW2-1-39-PEG to extract CTGF from the plasma of mice and then subjected them to Western blotting detection using an anti-CTGF antibody. As illustrated, AptW2-1-39-PEG bound to murine CTGF, which was further verified using the SPR assay (**Figure 5A**), and maintained stabilization in the murine plasma likewise (**Figure 5B**). Before treating the CIA mice with AptW2-1-39-PEG, we also evaluated its immunogenicity by treating the PBMCs from healthy people and healthy DBA/1 mice with aptamers. The results showed that even up to 2 µM of aptamer caused no significant difference in inflammatory cytokine concentrations in the cell medium (**Figures S5A, B**). Similarly, there was no significant difference in serum IgM and IgG levels between the treated and control mice (**Figure S5C**). In summary, AptW2-1-39-PEG showed low immunogenicity.

Morphologic observation of the CIA mice showed that AptW2-1-39-PEG-treated mice exhibited less severity of paw swelling (**Figure 5E**) and were ranked at lower arthritis clinical scores (**Figure 5D**). Consistently, knee joint injury was relieved by AptW2-1-39-PEG based on less inflammatory-cell infiltration, alleviation of synovial hyperplasia, and mitigatory bone destruction per H&E staining. Meanwhile, AptW2-1 restrained the proliferation of fibroblast in the synovium according to the IHC assay (**Figures 5E, F**). Furthermore, AptW2-1-39-PEG reduced the concentrations of the pro-inflammatory cytokines IL-1β, TNF-α, and IL-6 while increasing the anti-inflammatory cytokine IL-10 in the serum of the CIA mice (**Figure 5G**).

Generally, the results above indicate that AptW2-1-39-PEG alleviated joint erosion and inflammation in the CIA model, suggesting the promising potential of AptW2-1-39-PEG for therapeutics in RA.

### TSP-1 domain mediated the binding between CTGF and AptW2-1-39-PEG

**Figure 6:**
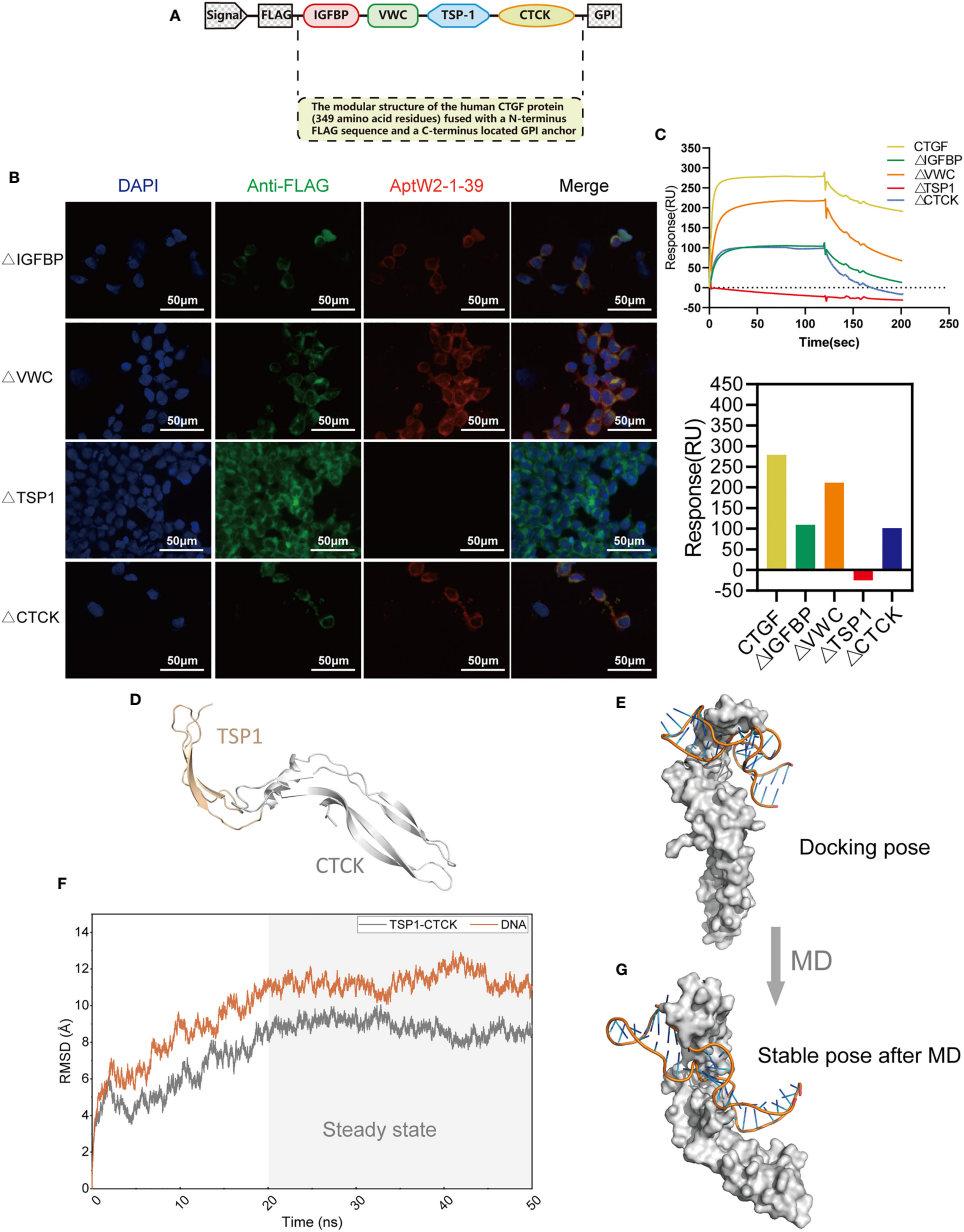
Identification of the binding domain of CTGF with AptW2-1-39-PEG. **(A)** The modular structure of human CTGF (349 amino acid residues) fused with an N-terminus FLAG sequence and a C-terminus located GPI anchor. **(B)** Immunofluorescence image of AptW2-1-39 and different deletion mutants of CTGF anchoring to HEK293T cells. The red color precisely indicates a Cy5-labeled AptW2-1-39-PEG. **(C)** SPR assay of AptW2-1-39-PEG targeting different deletion mutants of CTGF. **(D)** The TSP1-CTCK protein structure was predicted using homology modeling. **(E)** The TSP1-CTCK/AptW2-1-39 complex was predicted using molecule docking. **(F)** RMSD of the non-hydrogen atoms as a function of MD time. **(G)** The stable pose of the TSP1-CTCK/AptW2-1-39 complex after MD.

CTGF consists of four domains: insulin-like growth factor binding protein-like (IGFBP), von Willebrand factor type C repeat (VWC), thrombospondin type 1 repeat (TSP1), and C-terminal cystine-knot (CTCK) modules (47). To intuitively determine the direct region of the CTGF that interacts with AptW2-1-39, we designed a series of different deletion mutants of CTGF that were anchored to the surface of the cell membrane *via* flanking on the C-terminus by GPI anchor sequence and N-terminus by FLAG sequence (**Figure 6A**). The anchoring of the deletion mutants of the CTGF to the HEK293T cell membrane surface was confirmed using anti-FLAG fluorescent protein staining (**Figure 6B**). Meanwhile, AptW2-1-39 was incubated with the cells anchored to the mutants above. It was found that AptW2-1-39 bound to the TSP-1 domain of CTGF most tightly (**Figure 6B**). This supports the inhibited function of AptW2-1-39 toward angiogenesis, since the TSP1 protein plays an important role in angiogenesis (48). Meanwhile, the SPR assay also showed the same result (**Figure 6C**).

To further reveal the recognition mechanism of AptW2-1-39/TSP1, we managed to replicate this combination at the microscopic level using computer simulation. Initially, we modeled TSP1-CTCK using the multitemplate homology modeling method (**Figure 6D**) and exhibited the preliminary binding mode of the TSP1-CTCK/AptW2-1-39 complex using the protein–DNA docking technique. It was clear that Aptw2-1-39 was bound to the TSP1 domain at the molecular level (**Figure 6E**). Subsequently, we further evaluated the stability of the TSP1-CTCK/AptW2-1-39 complex under physiological conditions using MD simulation. Root mean square deviation (RMSD), describing the deviation of a system relative to the initial structure, is a significant parameter for evaluating the convergence of a system. As shown, the RMSD value fluctuated greatly in the early stage and subsequently came to convergence at 20 ns, which indicates the stabilization of complex conformation (**Figure 6F**). In this regard, we selected the conformation of the TSP1-CTCK/AptW2-1 complex at the 50-ns moment for further analysis. Surprisingly, we observed a package phenomenon: TSP1 was mostly wrapped by AptW2-1-39 in a semi-enveloping posture (**Figure 6G**). This suggested a strong binding force within the complex, thereby blocking the angiogenic activity induced by TSP1.

Overall, the results above suggest that the TSP-1 domain mediated the binding between CTGF and AptW2-1-39.

## Discussion

To date, the majority of therapeutic aptamers are still in the preclinical or early stage of clinical development for eye diseases, cardiovascular diseases, tumors, and inflammation (49). For example, pegaptanib, which was the first aptamer developed for wet age-related macular degeneration, was approved by the FDA, EMA, and PMDA in 2004, 2006, and 2008, respectively (50). Similarly, three newly generated aptamers—namely, AS1411, NOX-A12, and AGRO100—are currently in clinical trial for tumor therapeutics (51). These advances indicate the promising prospects for aptamers as therapeutic vehicles for certain troublesome diseases, including RA.

However, the development of clinically effective therapeutic aptamers has lagged far behind that of therapeutic antibodies, which could be attributed to the following reasons: First, aptamers are oligonucleotides that are easily degraded by nucleases. In addition, aptamers are filtered easily and excreted rapidly by the kidney due to their small diameters. Furthermore, their conformations may change, thus affecting their affinities or pharmacokinetics when applied *in vivo* (52). To minimize the adverse defects above, we employed a series of strategies during the in-SELEX and post-SELEX procedures, which are highlighted below. Firstly, choosing a DNA instead of an RNA aptamer as the objective. Although RNA aptamers have more diverse 3D conformations and stronger intra-strand RNA–RNA interactions that may increase binding affinity and specificity, RNAs are vulnerable to nuclease-mediated degradation, an unavoidable problem in a natural biological environment (53), which seems adverse when exposed to an inflammatory microenvironment of RA. Secondly, introduction of a counter-elution in SELEX using serum from healthy people. In a purified protein-based SELEX procedure (54), one of the most critical steps is selection partitioning, in which counter-elution excludes sequences that recognize other targets by using analog targets, thus increasing the selectivity of the aptamers (55). In this study, we deliberately added the counter-SELEX step using serum samples from healthy people to further improve disease specificity. Thirdly, employment of structural optimization post-SELEX.

Based on the strategies above, we successfully generated a novel aptamer AptW2-1-39-PEG with high affinity (KD 7.86 nM) targeting CTGF, which was characterized thoroughly for its satisfactory sensitivity (minimum protein binding concentration, 2 ng), specificity (negative binding to other biomarkers of RA), and stability (viability-maintaining duration in human serum, 48 h) properties using various biochemical and biophysical assays.

To guarantee the feasible application of the obtained aptamers in case of conformational changes in the physiological environment, we proceeded to assess the function and therapeutic effect of the obtained aptamers using functional experiments and animal models. This advanced our study into therapeutic application, differentiating it from two previous studies (34, 35). As expected, AptW2-1-39-PEG inhibited the proliferation of HUVECs and suppressed the activity of the angiogenesis induced by the CTGF verified both *in vitro* and *in vivo*. Importantly, the CIA murine model (a classical model of RA) was introduced to evaluate the therapeutic effect of AptW2-1-39-PEG on RA progression. Notably, AptW2-1-39-PEG relieved the severity of arthritis as well as inflammatory response in the CIA mice based on morphologic observation, histopathology evaluation, and serological test. These results guaranteed the functionality and suggested the promising potential of AptW2-1-39-PEG for RA therapeutics, which remains to be further explored in clinical studies.

The above results led us to further explore the direct combination pattern between AptW2-1-39 and CTGF. We intuitively revealed that the binding within AptW2-1-39/CTGF was mediated by the TSP1 domain of CTGF, which supported the suppressive function of AptW2-1-39 toward angiogenesis. Moreover, the surprising observation of a package phenomenon further confirmed the strong binding force within this complex.

Taken together, we obtained a novel DNA aptamer targeting CTGF and confirmed its qualified detection efficiency for diagnostics and its potential to suppress pannus formation for RA therapeutics.

## Data availability statement

The raw data supporting the conclusions of this article will be made available by the authors, without undue reservation.

## Ethics statement

The studies involving human participants were reviewed and approved by the Clinical Research Ethics Committees of the First Affiliated Hospital of WenZhou Medical University. The patients/participants provided their written informed consent to participate in this study. The animal study was reviewed and approved by Institutional Animal Care and Use Committee of WMU.

## Author contributions

GW performed the SELEX experiments and aptamer structural optimization. CL drafted and revised the manuscript. GW, BC, and ZC performed the endothelial tube formation and CAM assays. GW, CL, BC, ZC, and HZ performed the *in vivo* animal experiments. HZ, BL, and SJ performed the molecule docking and MD simulation. GW, CL, BL, and SJ conducted the statistical analysis. GW, CaL, XY, ChL, and JW conceived the study and participated in its design and coordination. GW and CL made equal contributions to this study. All the authors read and approved the final manuscript.

## Funding

This project was supported by the Key Research and Development Program of Zhejiang Province (Grant No. 2019C03023), the Zhejiang Provincial Natural Science Foundation (Grant No. LQ20H060003), the National Natural Science Foundation of China (Grant No. 81971539 and 82102534), and the Zhejiang College Students Innovative Entrepreneurial Training Program (Grant No. 2020R413005).

## Conflict of interest

The authors declare that the research was conducted in the absence of any commercial or financial relationships that could be construed as a potential conflict of interest.

## Publisher’s note

All claims expressed in this article are solely those of the authors and do not necessarily represent those of their affiliated organizations, or those of the publisher, the editors and the reviewers. Any product that may be evaluated in this article, or claim that may be made by its manufacturer, is not guaranteed or endorsed by the publisher.

## Supplementary material

Supplementary Figure S1 Top 100 sequences from the final aptamer products based on HTS analysis.

Supplementary Figure S2 Six groups of highly similar sequences sorted out from HTS analysis.

Supplementary Figure S3 SPR assay of 18 strands of ssDNA and AptW1-2. **(A)** SPR assay of 18 strands of ssDNA. **(B)** SPR assay of AptW1-2 targeting CTGF.

Supplementary Figure S4 Property assays and functional verification of AptW1-1-PEG. **(A)** Sensitivity assay of AptW1-1-PEG. **(B)** Specificity assay of AptW1-1-PEG. **(C)** Stability assay of AptW1-1-PEG incubated with plasma from healthy people. **(D, E)** Three-dimensional tube formation assay. HUVECs were treated with CTGF (100 ng/mL) and AptW1-1-PEG (500 nM) for 6 h. **(D)** Tube formation was observed and photographed. **(E)** The number of intersection nodes was calculated in the whole field.

Supplementary Figure S5 Immunogenicity assessment of AptW2-1-39-PEG. **(A, B)** Peripheral blood mononuclear cells (PBMCs) from healthy people were treated with AptW2-1-39-PEG (0 µM, 0.5 µM, 1 µM, and 2 µM) for 24 h. TNF-α and IFN-α in cell culture supernatants were quantified using ELISA kits. (n = 3 per group). **(C)** Serum total IgM and IgG levels of healthy DBA/1 mice on day 14 and day 28 after injection with APTW2-1-39-PEG. (n = 6 per group). The data are presented as mean ± SD.
