# Supplementary figures and images for: Connective tissue growth factor-targeting DNA aptamer suppresses pannus formation as diagnostics and therapeutics for rheumatoid arthritis
Source: Front Immunol. 2022 Aug 5;13:934061. doi: 10.3389/fimmu.2022.934061 (PMC9389230; doi:10.3389/fimmu.2022.934061)

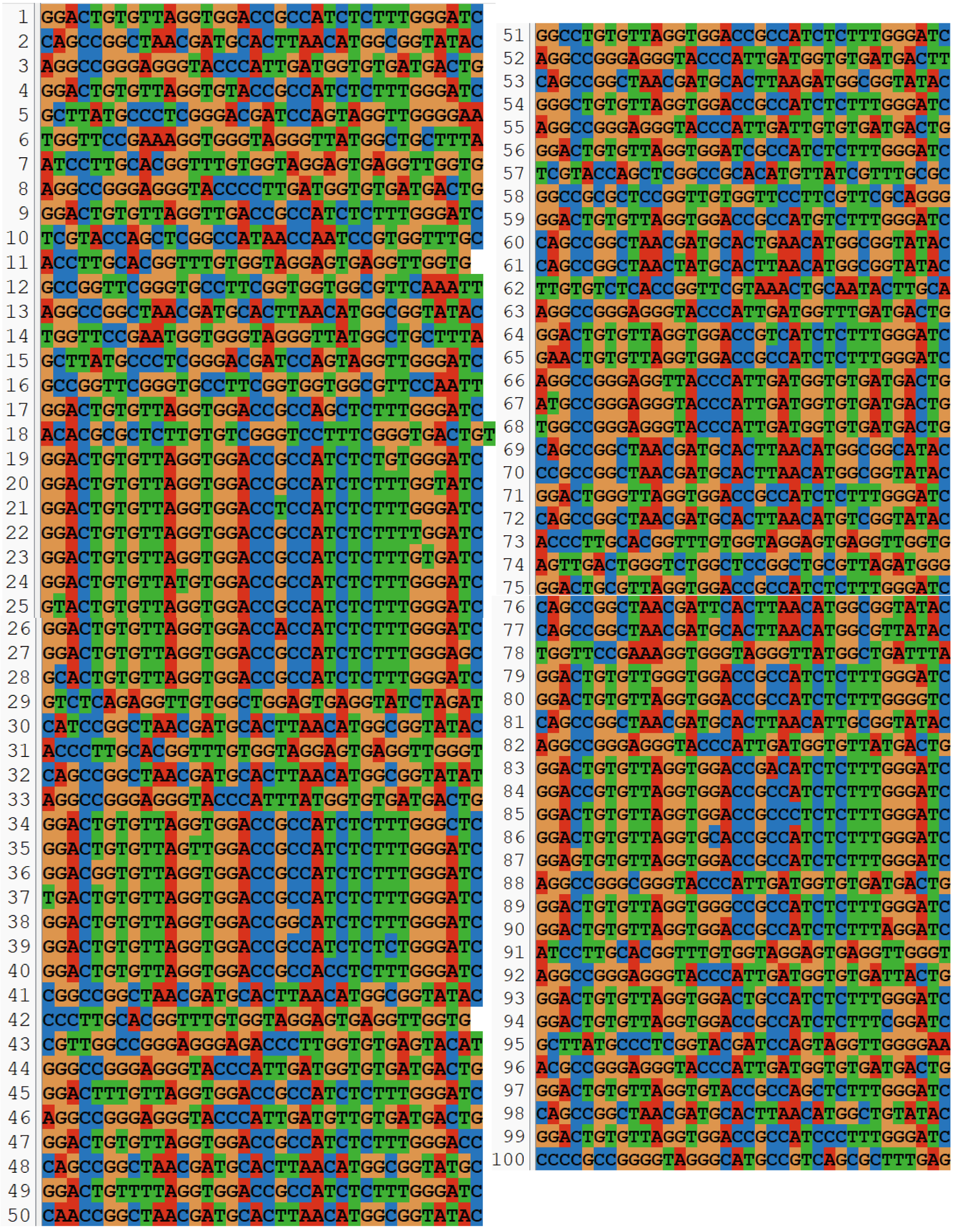

Supplement: Supplementary Figure S1 — Top 100 sequences from the final aptamer products based on HTS analysis. [file Image_1.tif]

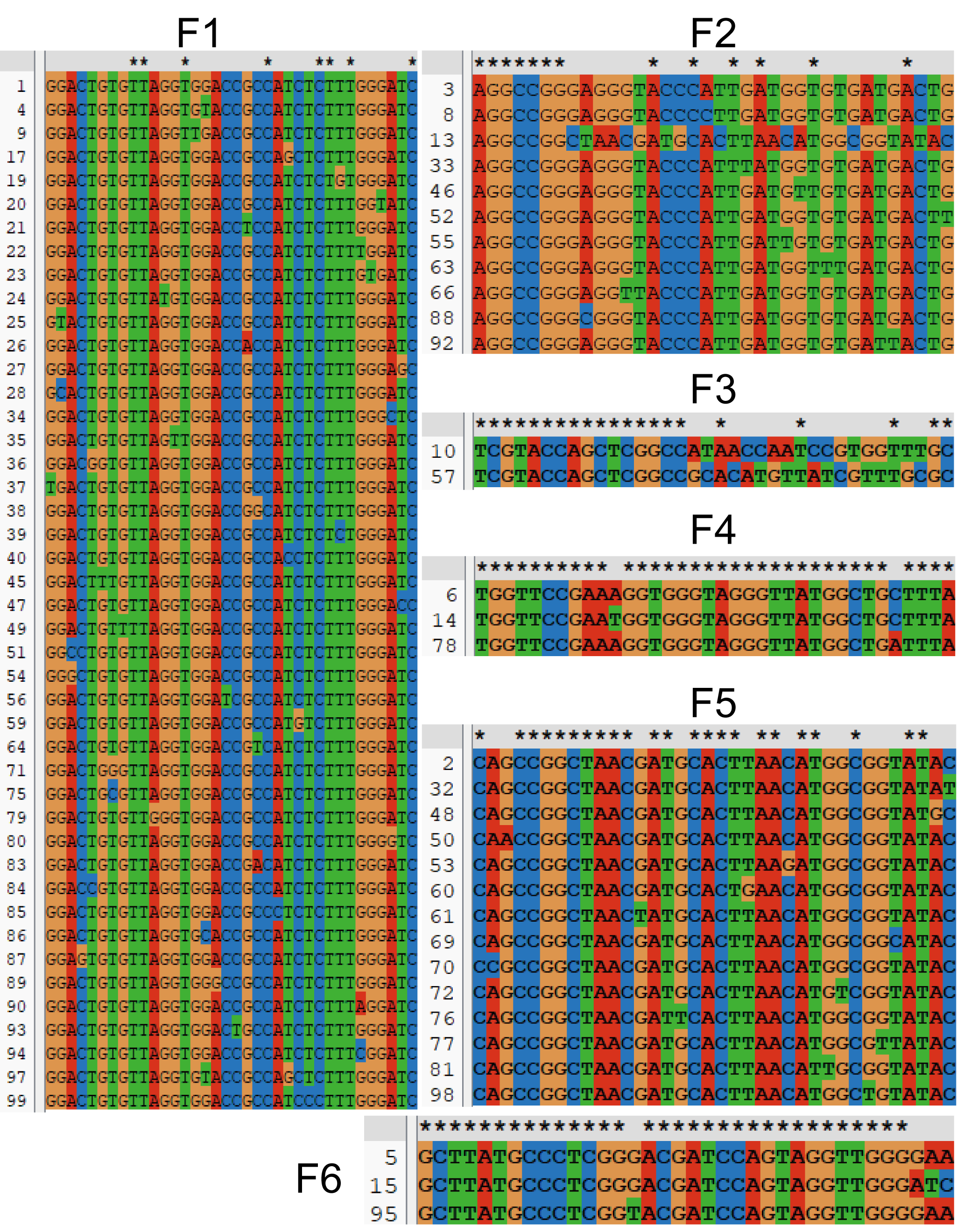

Supplement: Supplementary Figure S2 — Six groups of highly similar sequences sorted out from HTS analysis. [file Image_2.tif]

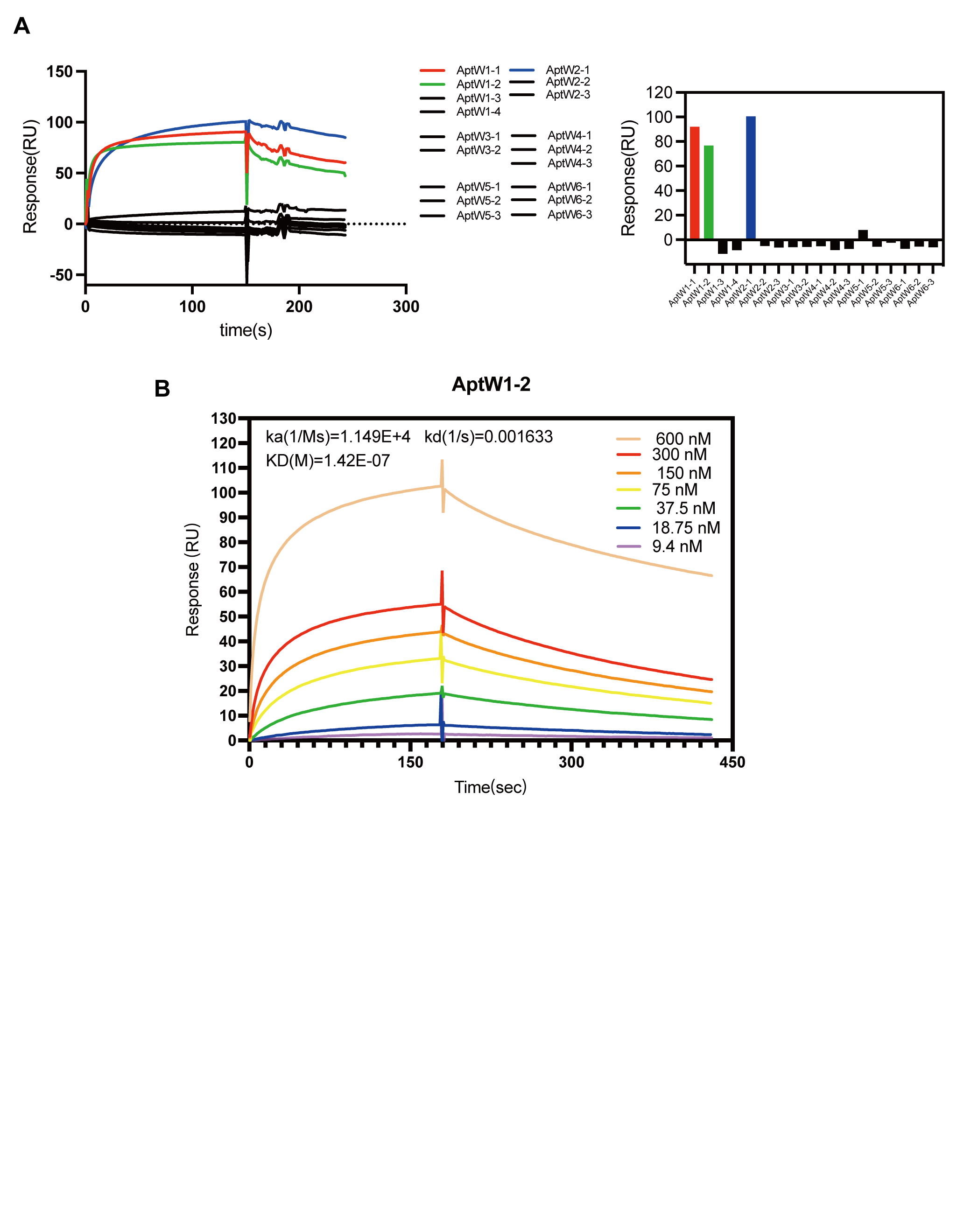

Supplement: Supplementary Figure S3 — SPR assay of 18 strands of ssDNA and AptW1-2. (A) SPR assay of 18 strands of ssDNA. (B) SPR assay of AptW1-2 targeting CTGF. [file Image_3.tif]

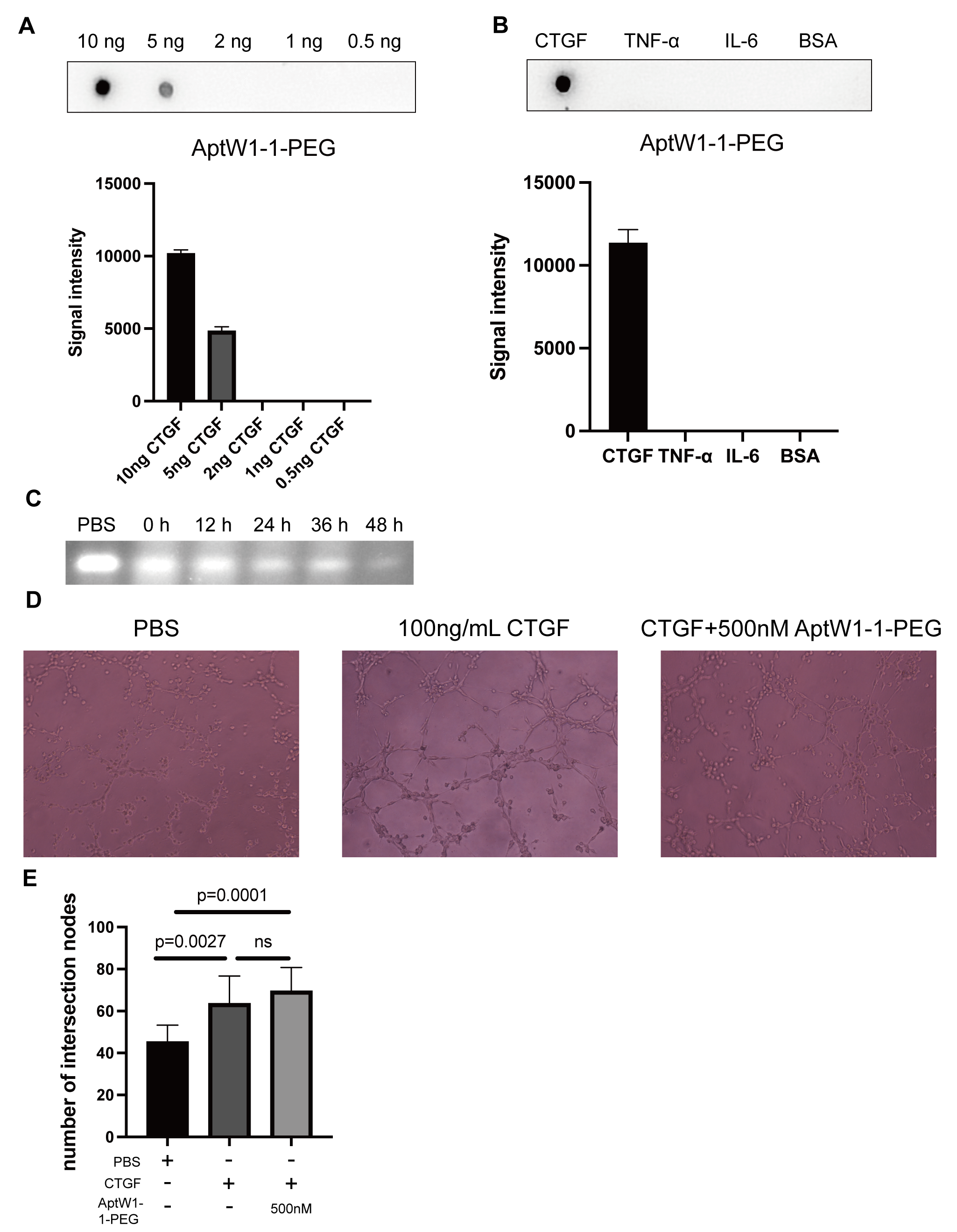

Supplement: Supplementary Figure S4 — Property assays and functional verification of AptW1-1-PEG. (A) Sensitivity assay of AptW1-1-PEG. (B) Specificity assay of AptW1-1-PEG. (C) Stability assay of AptW1-1-PEG incubated with plasma from healthy people. (D, E) Three-dimensional tube formation assay. HUVECs were treated with CTGF (100 ng/mL) and AptW1-1-PEG (500 nM) for 6 h. (D) Tube formation was observed and photographed. (E) The number of intersection nodes was calculated in the whole field. [file Image_4.tif]

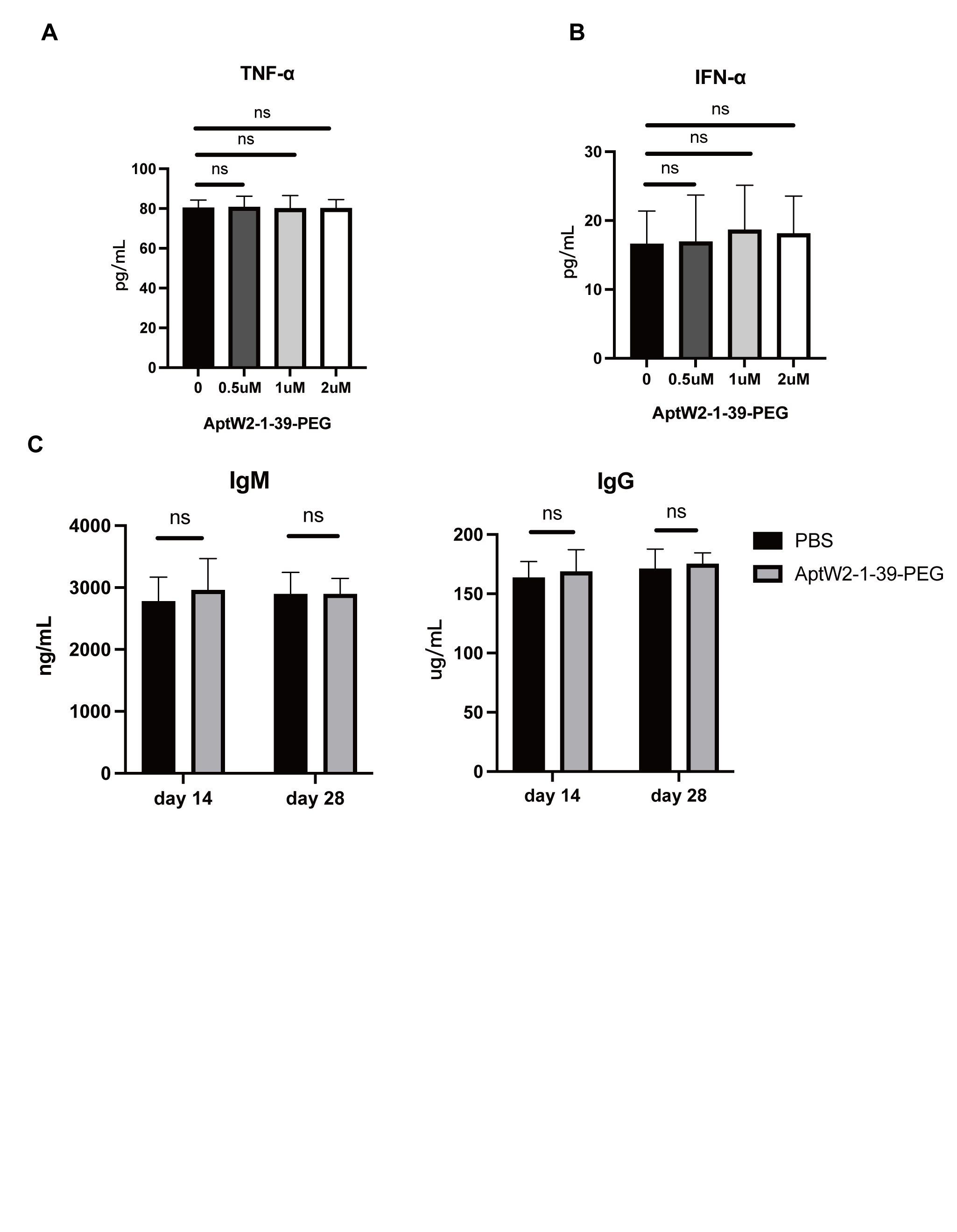

Supplement: Supplementary Figure S5 — Immunogenicity assessment of AptW2-1-39-PEG. (A, B) Peripheral blood mononuclear cells (PBMCs) from healthy people were treated with AptW2-1-39-PEG (0 µM, 0.5 µM, 1 µM, and 2 µM) for 24 h. TNF-α and IFN-α in cell culture supernatants were quantified using ELISA kits. (n = 3 per group). (C) Serum total IgM and IgG levels of healthy DBA/1 mice on day 14 and day 28 after injection with APTW2-1-39-PEG. (n = 6 per group). The data are presented as mean ± SD. [file Image_5.tif]
